# Management of hepatocellular carcinoma: An update

**DOI:** 10.1002/hep.24199

**Published:** 2011-03

**Authors:** Jordi Bruix, Morris Sherman

**Affiliations:** 1 Barcelona Clinic Liver Cancer (BCLC) Group, Liver Unit, Hospital Clínic, University of Barcelona, Institut d'Investigacions Biomèdiques August Pi i SunyerBarcelona, Spain; 2 University of Toronto and University Health NetworkToronto, Canada

Since the publication of the American Association for the Study of Liver Diseases (AASLD) practice guidelines on the management of hepatocellular carcinoma (HCC) in 2005, new information has emerged that requires that the guidelines be updated. The full version of the new guidelines is available on the AASLD Web site at http://www.aasld.org/practiceguidelines/Documents/Bookmarked&percnt;20Practice%20Guidelines/HCCUpdate2010.pdf. Here, we briefly describe only new or changed recommendations.

## Surveillance and Diagnosis

**Fig. 1:**
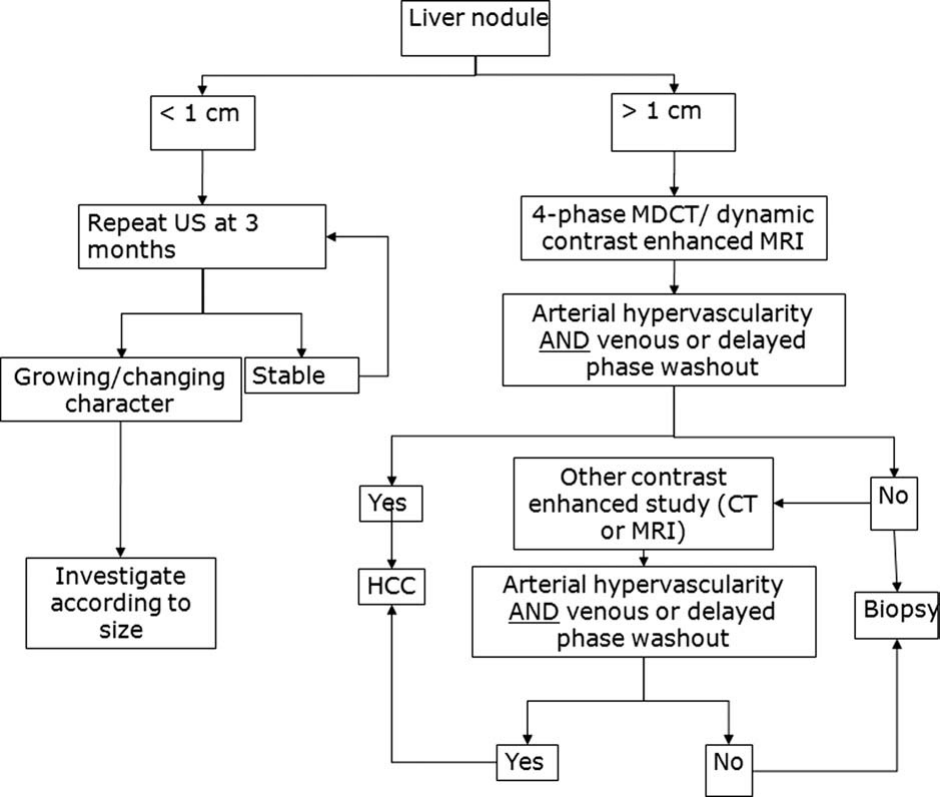
Diagnostic algorithm for suspected HCC. CT, computed tomography; MDCT, multidetector CT; MRI, magnetic resonance imaging; US, ultrasound.

In the previous guideline, groups were specified for which surveillance was likely to be cost-effective because the hepatocellular carcinoma (HCC) incidence was high enough. New data on defining HCC risk have emerged for hepatitis B virus,^1^,^2^ hepatitis C virus,^3^ and autoimmune hepatitis.^4^ Surveillance is deemed cost-effective if the expected HCC risk exceeds 1.5% per year in patients with hepatitis C and 0.2% per year in patients with hepatitis B. Analysis of recent studies show that alpha-fetoprotein determination lacks adequate sensitivity and specificity for effective surveillance (and for diagnosis).^5^,^6^ Thus, surveillance has to be based on ultrasound examination. The recommended screening interval is 6 months. Diagnosis of HCC should be based on imaging techniques and/or biopsy.The 2005 diagnostic algorithm has been validated and the diagnostic accuracy of a single dynamic technique showing intense arterial uptake followed by “washout” of contrast in the venous-delayed phases has been demonstrated.^7^-^9^ Contrast-enhanced US may offer false positive HCC diagnosis in patients with cholangiocarcinoma and thus, has been dropped from the diagnostic techniques. The diagnostic algorithm is shown in Fig. 1. The application of dynamic imaging criteria should be applied only to patients with cirrhosis of any etiology and to patients with chronic hepatitis B who may not have fully developed cirrhosis or have regressed cirrhosis. Interpretation of biopsies and distinction between high-grade dysplatic nodules and HCC is challenging. Expert pathology diagnosis is reinforced by staining for glypican 3, heat shock protein 70, and glutamine synthetase, because positivity for two of these three stains confirms HCC.^10^

## Staging and Treatment of HCC

**Fig. 2:**
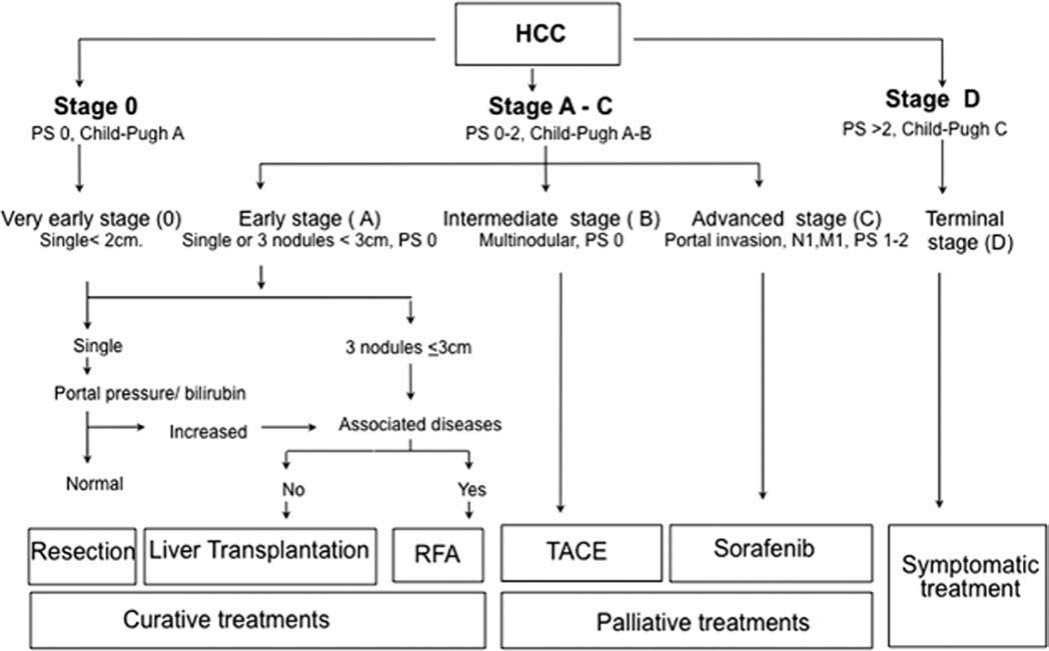
The BCLC staging system for HCC. M, metastasis classification; N, node classification; PS, performance status; RFA, radiofrequency ablation; TACE, transarterial chemoembolization.

The BCLC staging system (Fig. 2)^11^ has come to be widely accepted in clinical practice and is also being used for many clinical trials of new drugs to treat HCC. Therefore, it has become the *de facto* staging system that is used.

The recommendations for liver transplantation have not changed. No new data have emerged that can be used to define a new limit for expanding the patient selection criteria. The usefulness of portal pressure measurement to predict the outcome of patients and define optimal candidates for resection has been validated in Japan.^12^ Thus, resection should remain the first option for patients who have the optimal profile, as defined by the BCLC staging system. Although resection can be performed in some of these patients with advanced liver disease, the mortality is higher and they might be better served by liver transplantation or ablation. A cohort study of radiofrequency ablation demonstrated that complete ablation of lesions smaller than 2 cm is possible in more than 90% of cases, with a local recurrence rate of less than 1%.^13^ These data should be confirmed by other groups before positioning ablation as the first-line approach for very early HCC.

The recommendations regarding patient selection and method of administration of chemoembolization are unchanged. Radioembolization, i.e., the intra-arterial injection of yttrium-90 bound to glass beads or to resin, has been shown to induce tumor necrosis, but there are no data comparing its efficacy to transarterial chemoembolization or to sorafenib treatment for those with portal vein invasion. However, for patients who have either failed transarterial chemoembolization or who present with more advanced HCC, new data indicates the efficacy of sorafenib (a multikinase inhibitor with activity against Raf-1, B-Raf, vascular endothelial growth factor receptor 2, platelet-derived growth factor receptor, c-Kit receptors, among other kinases) in prolonging life.^14^,^15^ Sorafenib induces a clinically relevant improvement in time to progression and in survival The magnitude of the improvement in survival compares with other established molecular targeted therapies for other advanced cancers, and the associated toxicity is easily managed without treatment-related mortality. The most frequent adverse events were diarrhea (sorafenib versus placebo: 11% versus 2%) and hand–foot skin reaction (sorafenib versus placebo: 8% versus <1%), fatigue, and weight loss. Sorafenib is now considered first-line treatment in patients with HCC who can no longer be treated with potentially more effective therapies.

In summary, in the past decade HCC has gone from being an almost universal death sentence to a cancer that can be prevented, detected at an early stage, and effectively treated. Physicians caring for patients at risk need to provide high-quality screening, proper management of screen-detected lesions, and provision of therapy that is most appropriate for the stage of disease.

## Abbreviations

AASLD: American Association for the Study of Liver Diseases
BCLC: Barcelona Clinic Liver Cancer
HCC: hepatocellular carcinoma
